# Poisoning by "Mustard" Gas

**Published:** 1920-09

**Authors:** E. H. E. Stack, Carey F. Coombs, R. Rolfe

**Affiliations:** Ophthalmic Surgeon, Bristol Royal Infirmary; Surgeon, Bristol Eye Hospital; Physician with charge of Out-Patients, Bristol General Hospital; Honorary Consultant in Cardio-Vascular Cases, Ministry of Pensions, S.W. Region


### POISONING BY "MUSTARD" GAS.

BY

E. H. E. STACK, F.R.C.S., Capt., R.A.M.C.(T.F.),
*Ophthalmic Surgeon, Bristol Royal Infirmary; Surgeon, Bristol Eye Hospital;*

CAREY F. COOMBS, M.D., F.R.C.P., Capt., R.A.M.C.(T.F.),
*Physician with charge of Out-Patients, Bristol General Hospital; Honorary Consultant in Cardio-Vascular Cases, Ministry of Pensions, S.W. Region;*

AND

R. ROLFE, B.A., M.B., B.Ch. Cantab.

THIS paper consists of three parts. The first, compiled in 1917 in France by two of us (E. H. E. S. and C. F. C.), was not published at the time because of military considerations. The second part, written by one of us (R. R.) describes experiences of accidents incidental to the manufacture of "mustard" gas. The third part deals with some of the sequelæ of "mustard" gas poisoning as observed by one of us (E. H. E. S.) up to the present time. We think that our remarks may be opportune, since the public prints tell us of "mustard" gas shells being emptied of their contents in different parts of the country. This fact, together with the possibility that the gas may have to be manufactured again some day, makes it necessary to draw the attention of the profession and of all concerned to the need for precautions in the handling of the compound.

### Part I.

On the night of July 25th, 1917, 40 patients were admitted to No. 56 General Hospital suffering from gas poisoning of a new type, and three nights later 27 more were admitted. Owing to the prevalence of eye symptoms among them they were principally under the care of one of us (E. H. E. S.), while the other (C. F. C.) was told off by Col. J. Paul Bush, C.M.G., O.C. No. 56 General Hospital, to collect general data. The earlier part of this paper is taken from our report to Col. Bush, presented on Aug. 3rd.

Of the first batch of 40 men all had been gassed at Armentières between the 21st and 24th July, the source of the gas being enemy shells in every case. The second batch all came from Ypres; the majority had been gassed between 12th and 16th July.

Taking the 67 men together, we found that 23 were gassed only while in the open, the same number only while in cellars or dug-outs, 4 were gassed both in the open and in dug-outs, while the remainder (7) were not sure about the source. Gas helmets were used in 53 instances, the remainder consisting of men gassed in their sleep, and others who stated that they received no orders to put on helmets. Most of those who put on helmets thought that they failed to protect because they were taken off too soon under the mistaken impression that the gas had disappeared.

Of the men gassed in dug-outs several stated that there was no adequate protection against gas. In others the shell either entered the dug-out or destroyed its protection.

An extraordinary series of similes was offered when the men were asked what the gas smelt like, the general impression being that it was a bad smell, and the most popular comparisons being to some kind of vegetable decay.

The symptoms produced by the gas (which are now well known) were those of irritation of eyes, nose and stomach. In all but two cases there was eye irritation with lachrymation as a first symptom. Unlike the effects of lachrymatory gas, however, these did not follow gassing until after an appreciable interval, varying from 1 to 48 hours, and averaging nearly 7½ hours. Symptoms of nasal irritation occurred in 62 cases and headache in 56. Fifty-seven of the men vomited or retched, the average interval between gassing and the onset of this symptom being about 6½ hours. The throat became sore in 59 cases, the earliest symptom being felt on an average about 16 hours after gassing. In addition to these there were skin symptoms, to be referred to presently.

When the patients were admitted the symptoms were as follows :—

Conjunctivitis was present in all except 3, amounting to no more than a pinkness in the majority. In a similar number lachrymation was still present, and severe and purulent in a few. Photophobia was complained of by 56. The cornea was blurred in 14 cases, much more in the first batch than in the second, because the latter had had time to clear up. According to the patients eye symptoms had been at their worst from 3 to 48 hours after gassing, 24 hours or more in the great majority. The fundus oculi was normal in all cases.

In more than half the voice was husky or lost. The larynx was normal in only 9 cases, the remainder showing catarrh of the arytenoids, with pinkness of the cords in a few, and reddening of the tracheal mucosa in several. In contrast with the eye symptoms, these laryngeal changes were as marked in the second as in the first batch of men, being apparently of a more enduring character than the eye inflammation. In a few cases scattered sibili were to be heard, but the chest complications were on the whole not severe. One man had a transient albuminuria, but otherwise the urine was normal.

In 49 cases skin lesions were present. These appeared at an interval ranging from a few hours to 14 days after gassing, the average date of appearance being almost 4 full days after. The lesions were of two kinds, inflammatory and pigmentary. Apparently these were two stages of the same process, as in many cases pigmentation developed in the inflammatory areas as the signs of irritation cleared up. In some, however, pigmentation developed without any obvious erythema preceding it, and in others again the inflammatory phenomena subsided without giving rise to much pigmentation.

The commonest type of inflammatory lesion was an itching, smarting erythema, confluent in the centre but blotchy at the periphery of the areas affected. In some cases bullæ formed in these areas, but more often the skin peeled in flakes, leaving a sore, weeping surface, or else the redness changed rather abruptly to pigmentation, the subjective symptoms disappearing. In several cases there were blisters with little or no erythema round them, chiefly on the hands and forearm. Several others again had an itching eruption of scattered reddish papules on the buttocks or the front of the chest.

The pigmentary changes varied from a spotty discolouration of small areas, of a *café au lait* tint, to a deep browning, almost blackening, of large areas, broken only by pink patches where flakes of epidermis had come off. The depth of the discolouration in several of these cases was most striking, also its rather rapid development at a period of as many as 12 days after gassing.

Apparently it tends to clear up, partly no doubt by reabsorption and partly by desquamation. The only mucous membrane in which pigmentary changes were observed was that of the glans penis.

The areas most often affected by the erythema were the scrotum and fork, the flexures of the elbows, and the axillæ; but it was also seen on the neck, the front of the chest, and even on the toes. The distribution of the pigmented areas was practically the same; but in several of the most pronounced cases it attacked the lower abdomen and groins, and also the neck, with greater intensity than in other areas.

To this summary of symptoms made from our first batches of cases it is necessary to add certain later impressions drawn from a longer experience of large numbers of cases in order to complete the clinical picture. In particular we must lay stress on the severity of the respiratory symptoms. From the larynx down to the smaller bronchioles severe suffocative swelling develops. In fatal cases, of which we have had but one, this leads to an increasing dyspnœa with fever and cyanosis, but surprisingly little in the way of physical signs. The clinical picture in these circumstances is much like that of the purulent bronchitis which has been so prevalent in our armies in the winter. As in these cases, there is profuse purulent sputum. The actual lesion appears to be a necrosis of the tracheal and bronchial mucosa, followed by secondary invasion by the bacterial inhabitants of the respiratory tract. In one case these lesions were followed by the development of an empyema at the right base, which was opened and drained with satisfactory results. In the vast majority of our cases there have been no symptoms of respiratory disease apart from cough, distressing and persistent at night, and dyspnœa, persisting into convalescence without obvious physical signs.

In some cases also the skin symptoms were very severe, consisting of large blisters which develop within 24 or 48 hours after exposure to the gas. Nearly always this is attributable to unusually direct contact with the toxic contents of the shells. In many cases the shell burst so near to the man that it is probable that its liquid contents were splashed on his skin or clothing. In several, again, the blisters were limited to the ankles, the men having been exposed as stretcher-bearers to shell-contents in shell-holes. In one instance these lesions were so widespread and severe that the patient died of shock and toxæmia as in an ordinary burn of corresponding degree.

There have been a few instances in which the gastric symptoms have been severe and persistent—vomiting and epigastric pain, with tenderness, increased by food, and not yielding quickly to treatment. We have seen no case in which these symptoms were at all threatening, but several in which they have persisted without the slightest improvement for over a week after admission.

In all but two points these symptoms are easily explicable. Eyes, nose, throat, bronchi and skin all bear the marks of severe injury by direct contact with some irritant. But this assumption does not get rid of two difficulties : first, why is there so long a latent period between the time of gassing and the time of the onset of the symptoms ? second, how are we to explain the pigmentation ?

To dispose of the latter point first. The pigment is not ' dirt,'' a deposit on the surface, for it is impossible to get it off by any cleansing agent. On the other hand, it is not a true pigment. It peels off with the cutis thrown off by desquamation ; further, sections of a portion of pigmented skin excised from one of our patients show that there is no increase of melanin in the skin. The only satisfactory explanation is that suggested by Capts. E. P. Cathcart and

B. J. Collingwood, who kindly came to see our cases, to the effect that the pigment is a metallic sulphide formed by interaction of the gas with chemical processes in the dying cuticle. This explains its rather sudden appearance, its obviously superficial position, and its disappearance with desquamation.

Two explanations of the latent period are available. According to the first, the effect of the gas on the conjunctivæ, skin and throat, bronchi and stomach is a direct irritation, delayed only because it depends on decomposition of the gas in contact with moisture. On this hypothesis it is to be presumed that the actual irritant is some breakdown product of the gas which in its turn enters into union with the tissues attacked. The other theory would assume absorption of the gas into the body, and re-excretion with injury to the skin and mucosæ as it and its by-products pass through on their way out. In favour of the latter is the fact that skin symptoms may develop many days after gassing, in spite of the patient's clothes having been changed within twenty-four hours after gassing. Against it and in favour of the direct irritation theory are these two points: first, that the poison when in sufficiently concentrated liquid form can raise blisters in the skin; and, second, that late erythema and pigmentation have been much less frequently encountered in those patients who were treated in Field Ambulances and elsewhere shortly after gassing with alkaline baths.

It may be added that we should expect to find a large number of cases with evidence of renal damage if the gas were absorbed into and re-excreted from the circulation. In point of fact, we have only seen one case of nephritis complicating "mustard" gas poisoning, and in this case the renal lesion had probably begun to develop before gassing occurred. Moreover, only one of our first 67 cases developed any albuminuria, and this only lasted for a day or two.

We have found 2 per cent. sodium bicarbonate lotion a very useful application for all lesions—eye, skin and throat.

As both erythema and pigmentation reminded us of arsenical poisoning we consulted Lieut. R. Gaunt, Anti-Gas Instructor, No. 2 Base Training Camp, who kindly gave us his expert opinion as to the possibility of arsenical compounds being employed in gas shells. He thought it was possible, and we therefore asked Capt. Maclean, R.A.M.C., working at No. 46 Stationary Hospital, to see if arsenic were present in the urine. Three of the severest cases were selected, and twenty-four hour specimens collected. Capt. Maclean was good enough to examine these for arsenic, but with negative result.

We express our thanks to Lieut.-Col. J. Paul Bush, C.M.G., R.A.M.C.(T.F.), and to our colleagues for permission to use these records.

### PART II.

The following notes give an account of the experiences of " mustard " gas poisoning which one of us (R. R.) gained while acting as unofficial medical adviser to a munition factory in 1918 during a period of several months. At this time the medical arrangements were in charge of a trained sister with two untrained assistants. Later on a whole-time medical officer was appointed.

It was in May or June that I was warned that a dangerous gas was about to be manufactured at the factory. On June 18th I was sent for to see one of the chemists who was suffering from eye trouble due to the gas. His symptoms were photophobia and lachrymation, and the eyes could scarcely be opened owing to spasm and œdema of the lids. There was severe conjunctivitis and intense pain, which was slightly relieved by 2 per cent. cocaine solution. The eyes were bathed with dilute boric lotion and bandaged. The room was darkened and frequent irrigation with warm weak glycothymoline was instituted. Later in the day an ophthalmic surgeon saw him with me and advised that we should continue to use weak glycothymoline and cocaine drops with atropine. He also suggested that red light might be found comforting. By that time definite iritis had developed. The following day the patient was somewhat easier, but the iritis was more marked and the cornea was roughened. Under the treatment outlined above the patient improved slowly, but the photophobia remained very obstinate. By the end of June he was able to go away for a rest. He was obliged to wear dark glasses in any but the most subdued light.

On July 7th six chemists were more or less severely gassed. Again the eyes suffered most, though they all had throat trouble with husky speech and cough. The eye symptoms were the first to appear, beginning 4 to 48 hours after exposure. Lachrymation and spasm of lids, due presumably to photophobia, were very intense, so that it was difficult to examine the eyes. The sister having been informed that 10 per cent. solution of sodium bicarbonate was the best lotion to use, this was employed every two hours as an eye-wash, but it was found necessary to use cocaine drops also to relieve the pain, which was present even with the eyes closed and bandaged. Red light did not seem to afford any relief. On July 9th an ophthalmic surgeon saw the patients with me and supported the plan of treatment. He warned me of the danger of perpetuating the blepharo-spasm if light were excluded too long and too completely, and said that it was very difficult to overcome. Every effort was therefore made to get the patients to bear exposure to light as soon as possible. The eye lesions were not so severe as in the first case, none of them showing more than quite slight iritis. The value of early exposure to light was, however, obvious, the photophobia disappearing much more quickly than in the first case. The ophthalmologist also told me that the corneal roughening was not likely to be permanent.

After this it became a routine procedure to wash out immediately the eyes of any person exposed to traces of gas with 10 per cent. solution of bicarbonate of soda. This involved a heavy strain on the sister, as she did nearly all this work personally.

No further mishap occurred for some time, but there were numerous cases of gas burns which the sister attended to herself. Her plan was to snip the blisters under hot water and to cut away all loose skin ; then to soak the affected part in water as hot as could be borne for some time, half an hour or more ; and afterwards to apply boric fomentations which were frequently changed. The hot bath was repeated twice daily. The results were on the whole good. There was, however, a distinct tendency for burns which had almost completely healed to relapse.

I saw chiefly men suffering from laryngitis causing husky voice, some pain, and dysphagia. Others had very severe cough, especially at night, with very slight physical signs of bronchitis. This cough was difficult to relieve, and I failed to find any drug or combination of drugs which could be relied on to do much good or to shorten the very protracted course of the trouble. Ipecacuanha wine was perhaps the most useful. Spraying or gargling did some good to the throat symptoms.

Another common complaint was pruritus. Often there was no apparent skin lesion except a few tiny red papules. These were chiefly on the feet and legs, and leather boots appeared to afford no protection from the gas. The most common site for pruritus, however, was the scrotum and folds of the groins, and here the skin became roughened and there was slight desquamation. I found that an ointment of zinc oxide with resorcin and ichthyol gave great relief, and sufferers came to me for some time after I ceased my connection with the factory to get this ointment. A very peculiar point was that men who had been affected on former occasions were much more readily injured by fumes than those who had not previously been exposed to them.

On August 10th eight men were gassed. The symptoms were chiefly respiratory, and four at least of the men were removed at once to hospital. I had little chance of studying these cases, as the men were so ill, gasping for breath, that no time was lost in removing them by ambulance. About this time a whole-time medical officer was appointed to the factory, and my connection with it came to an end.

If in the future this gas should have to be handled, whether for its manufacture or its destruction, the utmost precautions should be taken to warn everyone against its deleterious effects. The nature of its effects should be explained to them, both to encourage them to seek early treatment and to dispel that atmosphere of mystery which is so potent a factor in the causation of the neuroses alluded to in Part III. Every facility should be given for immediate treatment of symptoms. We think medical officers responsible for these procedures should be fully informed as to the best treatment. They might well be supplied, for example, with such publications of the Medical Research Council as bear on the subject.

### Part III.

At the time of seeing these cases during the first few weeks one thought that they would clear up quickly and leave no after-effects, but this idea was dispelled. They turned out in a small percentage of cases to be very troublesome. The condition became a persistent or intermittent photophobia and lachrymation with almost no injection, mixed up with a marked neurosis, and the treatment had to be directed much more to this latter element than any other.[1]

Continuous reassurance of perfect recovery with some sort of faith cure had to be the line of treatment ; avoidance of shades and dark glasses with cold lotions and occasional astringents, in the end brought about or accompanied a cure.

Many cases showed the usual mixture of sub-conscious malingering and hypochondria, but one is seeing as time goes on fewer and fewer of these amongst the pensioners.

### Progress of the Medical Sciences.

[1] This is on all fours with our experience of the "irritable heart" that sometimes persists after gassing. Nine-tenths of it is due to psychoneurosis, and it is this element that offers most possibility of treatment (C. C.).

